# Selective Labelling of Cell-surface Proteins using CyDye DIGE Fluor Minimal Dyes

**DOI:** 10.3791/945

**Published:** 2008-11-26

**Authors:** Asa Hagner-McWhirter, Maria Winkvist, Stephanie Bourin, Rita Marouga

**Affiliations:** Research and Development, GE Healthcare Bio-Sciences AB

## Abstract

Surface  proteins are central to the cell's ability to react to its environment and to interact with neighboring cells. They are known to be inducers of almost all intracellular signaling. Moreover, they play an important role in environmental adaptation and drug treatment, and are often involved in disease pathogenesis and pathology (1). Protein-protein interactions are intrinsic to signaling pathways, and to gain more insight in these complex biological processes, sensitive and reliable methods are needed for studying cell surface proteins. Two-dimensional (2-D) electrophoresis is used extensively for detection of biomarkers and other targets in complex protein samples to study differential changes. Cell surface proteins, partly due to their low abundance (1 2% of cellular proteins), are difficult to detect in a 2-D gel without fractionation or some other type of enrichment. They are also often poorly represented in 2-D gels due to their hydrophobic nature and high molecular weight (2). In this study, we present a new protocol for intact cells using CyDye  DIGE Fluor minimal dyes for specific labeling and detection of this important group of proteins. The results showed specific labeling of a large number of cell surface proteins with minimal labeling of intracellular proteins. This protocol is rapid, simple to use, and all three CyDye DIGE Fluor minimal dyes (Cy 2, Cy 3 and Cy 5) can be used to label cell-surface proteins. These features allow for multiplexing using the 2-D Fluorescence Difference Gel Electrophoresis (2-D DIGE) with Ettan  DIGE technology and analysis of protein expression changes using DeCyder  2-D Differential Analysis Software. The level of cell-surface proteins was followed during serum starvation of CHO cells for various lengths of time (see Table 1). Small changes in abundance were detected with high accuracy, and results are supported by defined statistical methods.

**Figure:**
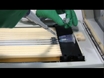


## Protocol

### Cell culture

1. Grow Chinese Hamster Ovary cells (CHO-K1) using standard cell culture procedures in F-12 Ham medium with GlutaMAX I containing 10% fetal calf serum, 50 U/ml penicillin, and 50 µg/ml streptomycin sulfate (Invitrogen).
2. Exchange the culture medium to serum-free media. Label the cell surface proteins were at different time points with CyDye DIGE Fluor Cy3 or Cy5 minimal dyes (see section *Cell Surface Labeling*, below).
3. Pool equal numbers of cells from each time point and label with CyDye DIGE Fluor Cy2. Use these as an internal standard for each 2-D gel.
4. The majority of the experiments can be performed with CHO-K1 cells, but mouse embryo fibroblasts (3T3 L1) and mouse ascites lymphoma lymphoblasts (EL4) can also used (data not shown).
5. Grow the two latter cell types in DMEM medium with GlutaMAX II, but, otherwise, use identical conditions used for the CHO-K1 cells.

### Cell surface labeling

1. Carefully detach adherent cells non-enzymatically, counting and dividing into aliquots of 5–10 x106 cells. For cells growing in suspension, omit the detaching step.
2. Centrifuge the cell suspensions at about 800 x g for 5 minutes. Remove the supernatants containing the medium.
3. Wash the pellets by resuspendion in 1 ml ice cold Hank’s Balanced Salt Solution (HBSS) pH 8.5. Centrifuged at 800 x g at 4°C for 2 minutes.
4. Remove the supernatant and resuspend the cell pellet in 200 µl ice cold labeling buffer (HBSS pH 8.5, 1 M urea).
5. Label the intact cells with 600 pmol CyDye DIGE Fluor minimal dyes for 20 minutes on ice in the dark.
6. Quench the reaction by adding 20 µl 10 mM lysine. Incubate for 10 minutes.
7. Wash the surface-labeled cells twice by resuspension in 500 µl HBSS pH 7.4, followed by centrifugation at 800 x g at 4°C for 2 minutes.

### Cell lysis and fractionation

1. Lyse the surface-labeled cells in 150 μl cold lysis buffer (7 M urea, 2 M thiourea, 4% CHAPS, 30 mM Tris, 5 mM magnesium acetate pH 8.5). Leave on ice for at least 1 h with occasional vortexing.
2. Centrifuge the lysates at 10 000 x g at 4°C for 5 minutes. Transfer the supernatant to a new tube. This sample is the non-fractionated sample containing all cellular proteins.
3. In parallel, wash cell pellets, as above, then fractionate (using a membrane fractionation kit, Pierce) into membrane and cytosolic fractions prior to 2-D gel electrophoresis. The membrane fraction contains internal and cell surface membrane proteins.
4. For comparison, follow the standard Ettan DIGE procedure ^3^, and lyse, label, and, finally, fractionate the cells. Determine the protein concentration in the samples using the 2- D Quant Kit (GE Healthcare).

### 2-D electrophoresis

1. Rehydrate Immobiline DryStrip gels, pH 3–11NL (24 cm) using Immobiline DryStrip Reswelling tray, 24 cm in 450 µl DeStreak Rehydration solution (0.5% IPG Buffer) overnight.
2. Apply the CyDye-labeled samples (corresponding to 50 µg total protein) to Immobiline DryStrip gels by anodic cup loading in the manifold and perform isoelectric focusing (IEF) using Ettan IPGphor II IEF System according to instructions.
3. After IEF, equilibrate the strips in two steps and place on top of large (26 x 20 cm) 12.5% polyacrylamide gels (SDS-PAGE). Overlay with 0.5% agarose (in running buffer containing bromophenol blue). Run 2-D electrophoresis using Ettan DALTtwelve Large Vertical System at 5 W/gel for 30 min, and then at 15 W/gel until the dye front reaches the bottom of the gel.

### Imaging and data analysis

1. After completing 2-D electrophoresis, scan the gels for Cy2, Cy3 or Cy5 using a Typhoon 9410 Variable Mode Imager.
2. Compare spot maps from membrane fractions, cytosolic fractions, and non-fractionated samples using DeCyder 2-D Differential Analysis Software ^4^.

### Post-staining

1. After imaging, silver stain the gels according to standard procedure ^5^.

### Protein identification

1. Grow, harvest, wash and lyse preparative amounts (approximately 1 mg total protein from 10 x106 CHO cells) of cells, as described above for a non-fractionated sample. Use a Cy5 cell surface labeled sample (see above) as a spike, and apply together with the unlabelled preparative amounts of protein.
2. Carry out 2-D electrophoresis as described above, but this time, aplly 600 µg unlabeled cell lysate together with 50 µg cell surface labeled spike by anodic cup application. Use reference markers to allow correct spot picking, and place between the glass plates before gel casting according to recommendations ^3^.
3. Scan the gel in Cy5 channel to obtain the Cy5 cell surface spot map, followed by total protein staining using Deep purple total protein stain ^3^.
4. Match together the two spot maps using the DeCyder 2-D software and create a pick list for all the spots corresponding to the Cy 5 labeled cell surface proteins. Pick cell surface proteins using the Ettan spot handling station, extract from the gel plugs, and trypsinate using the Ettan digester. Identify using MALDI-TOF mass spectrometry.

**Table 1.** An Ettan DIGE experiment was performed using samples from serum depleted cells labeled according to the cell-surface protein labeling protocol in figure 1.

**Table:** 

Sample	Time of serum depletion	**Labelled with CyDye**	Gel number
1	-	Cy 3, Cy 2	1
2	30 minutes	Cy 5, Cy 2	1
3	2 hours	Cy 3, Cy 2	2
4	4 hours	Cy 5, Cy 2	2
5	16 hours	Cy 5, Cy 2	3

 

## Discussion

### Protein concentration

An overview of the two labeling workflows is shown in Figure 1. Since the cells are still intact when labeled according to the cell-surface protein labeling protocol, only the cell surface proteins are exposed to the dye. In the standard Ettan DIGE protocol, the cells are lysed before labeling and proteins inside as well as outside the cell are labeled (Fig 1). The relative amount of dye to protein in the cell-surface protein labeling protocol is not known, since cell-surface proteins cannot be specifically quantitated. However, it is known that cell surface proteins constitute a very low proportion of the cellular proteins ^2^. Approximately 5–10 x10^6^ cells to 600 pmol of dye were used. It may be possible to use fewer cells since only 12.5–25% of the nonfractionated sample in this study was used for 2-D electrophoresis.


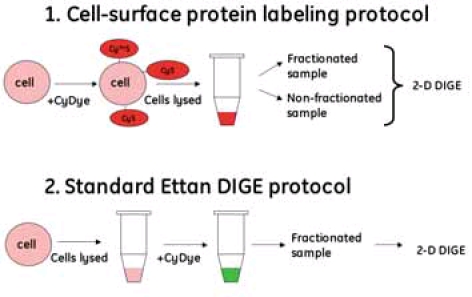


### Fig 1. Overview of labeling workflow protocols

Protein concentrations in the different fractions were determined using the 2-D Quant Kit. The total protein amount derived from 10 x10^6^ CHO-K1 cells was 920 μg in the non-fractionated sample, 225 μg in the membrane/hydrophobic fraction and 770 μg in the cytosolic/hydrophilic fraction. These amounts will most likely vary depending on cell type and cell size. The proportion of proteins that are labeled in the cell-surface protocol may be higher than the standard Ettan DIGE minimal labeling, which is 2-3% of total protein. It seems that only one dye molecule is attached per protein molecule, since the spot shape is round and vertical streaking of the low molecular proteins on the gels is absent (Fig 2 and 3). Two and more dye molecules per protein would cause vertical streaking due to increased molecular weight of the labeled protein. This phenomenon would mostly be seen with the low molecular weight proteins, since a change in molecular weight of these proteins would result in a larger shift on the gel compared to high molecular weight proteins.

Cell-surface protein specific labeling

Two identical samples of cells were surface labeled with CyDye DIGE Fluor Cy3. One was lysed and used directly for 2-D electrophoresis. The other sample was lysed and fractionated into membrane and cytosolic fractions. The entire fluorescent label appeared in the membrane fraction; the cytosolic fraction was devoid of any labeled proteins (Fig 2). The same gel with the cytosolic protein sample was silver stained and the result showed that there were proteins in the gel, but they were not labeled using the cell-surface protein labeling protocol. These results suggest that this new labeling protocol is specific for cell surface proteins. The CyDye DIGE Fluor minimal dye does not appear to enter the cell or pass through the cell membrane. The cells are kept on ice prior to the labeling and this may reduce any transport across the membrane. The time for CyDye DIGE Fluor minimal dye exposure is also relatively short (20 minutes), which is sufficient for protein labeling but not for entry into the cell. Another possible explanation for the lack of labeling inside the cell is that even if the dye passes across the membrane, the pH inside the cell is too low (< pH 7.4) for an efficient labeling reaction to occur (optimal pH 8.5). The labeling reaction is quenched followed by washing of the cells, which further prevents any protein labeling after the cells have been lysed. This method has been successfully applied to human cell lines in vitro as well as to a complex biological system in vivo ^7^.


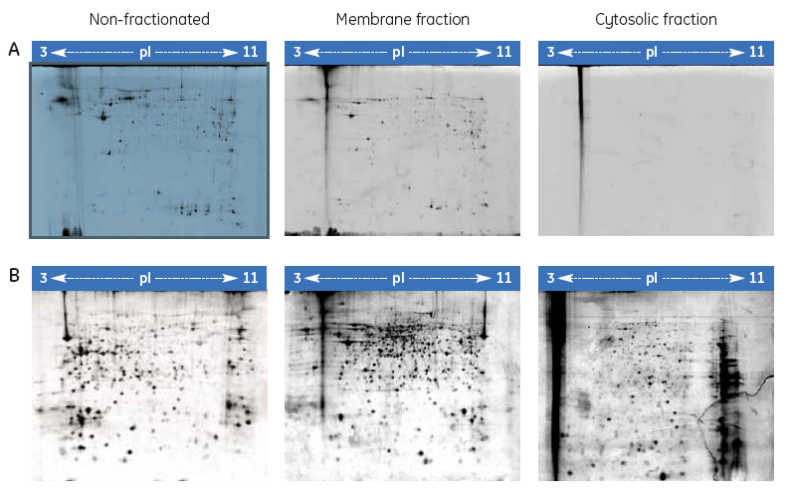


### Fig 2. Specificity of cell-surface protein labeling.

The cellsurface proteins of CHO-K1 cells were labeled with Cy3 and fractionated. The different fractions were separated by 2-D electrophoresis and scanned for Cy3 fluorescence (A). The same gels were then silver stained (B).

### Fractionation

There are only minor differences in the spot pattern for the membrane-fractionated sample compared with the nonfractionated sample (Fig 2). The two spot maps were compared using DeCyder 2-D Differential Analysis Software and all the spots detected in the membrane fraction were also present in the non-fractionated sample. Fractionation, therefore, is not necessary to improve detection of cell surface proteins but can be used to verify lack of labeling of proteins inside the cells.

### Comparison between protocols

To be able to evaluate the advantages with the new cell surface protein labeling protocol, a comparison with the standard Ettan DIGE protocol was performed. Two identical samples from CHO-K1 cells grown in the same flask were labeled in parallel with the two different protocols, respectively (Fig 1). A cell-surface Cy5 labeled sample was run on the same gel as a Cy3 labeled cell lysate. The green spots (Cy3) on the gel (Fig 3A) represent the proteins labeled using the standard Ettan DIGE procedure followed by membrane fractionation. These spots are presumably membrane proteins including cell surface proteins as well as proteins from membranes inside the cell (ER, Golgi, mitochondrion, and nucleus). Standard Ettan DIGE labeling procedure followed by a membrane fractionation step was chosen for comparison, since it should give the highest probability for detecting the low abundant cell surface proteins. The red spots (Cy 5) on the gel (Fig 3A) are cell surface specific proteins labeled using the new cell surface protein labeling protocol that are not visible with the standard labeling procedure (green spots). Furthermore, the yellow spots represent overlapping proteins that occur in both samples using either procedure (Fig 3A). Another useful application would be to combine both labeling protocols to distinguish proteins on the cell surface from those on intracellular membranes. Moreover, information about relative changes in cell surface/membrane protein levels after various stimuli could be followed by using both labeling techniques simultaneously.


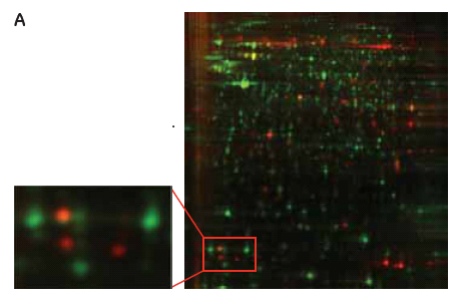
 
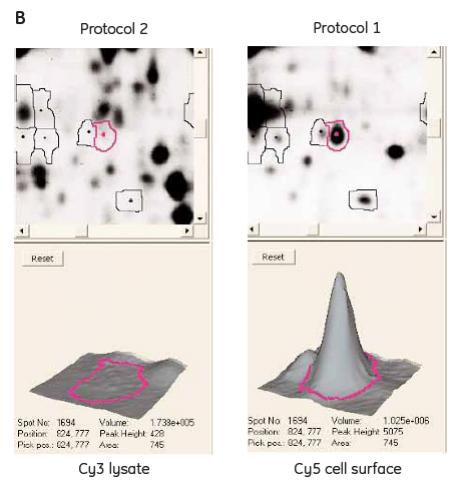


### Fig 3.

(A) 2-D gel images of a CHO-K1 Cy5 cell-surface labeled sample (red spots, see protocol 1, Fig 1) and a membrane fractionated Cy3 sample (green spots, see protocol 2, Fig 1) labeled according to standard Ettan DIGE protocol run in the same 2-D gel. (B) DeCyder 2-D Differential Analysis Software views from the 2-D gel showing a cell-surface labeled protein not visible using the standard Ettan DIGE protocol.

### Identification of cell surface proteins

Since the spot pattern is very different between a cell surface labeled sample and a total protein labeled sample, it was necessary to include a cell surface labeled spike to enable matching and identification of the cell surface spots in the preparative spot map. All cell surface proteins were picked. Cell surface proteins can be difficult to identify due to their low abundance. The actual protein amount in some spots may be insufficient for identification, since the cell surface proteins are visually enriched and not physically enriched using this protocol. To facilitate successful identification of low abundant cell surface proteins, the preparative amounts of total protein can be enriched for membrane proteins before application on 2-D electrophoresis. In these CHO cells, intracellular membrane proteins and cell surface proteins constitute approximately 20% of the total protein in the cells. For this cell type, the protein amount in the spots could potentially be increased by a factor 5 by enrichment of membrane proteins. Also, the use of narrow gradient pH intervals of the IPG strips will allow application of larger amounts of protein. In this study we used only 600 μg total protein, with no enrichment for membrane proteins, and a broad range IPG strip. We were still able to identify a large number of cell surface proteins, of which 82% were previously known as membrane associated proteins.

### Multiplexing

To test the cell surface protein labeling protocol in an Ettan DIGE experiment using all three dyes ^6^, a series of samples from serum depleted CHO cells were collected and cell surface proteins labeled at different time points (Table 1). Samples were separated by 2-D electrophoresis. The preparation of an internal standard for a cell surface DIGE experiment is straightforward. In this case, Cy 2 cell surface labeled samples (from all time points) were pooled and used as an internal standard applied to each 2-D gel. All three CyDye DIGE Fluor minimal dyes labeled cell surface proteins similarly (data not shown). Changes in expression during serum starvation for many of cell surface proteins were detected using DeCyder 2-D software (Fig 4).


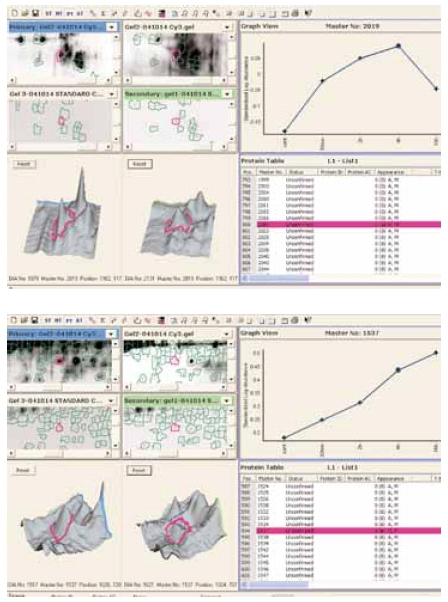


### Fig 4.

Change in expression of two cell-surface proteins during starvation of CHO-K1 cells. Spot maps were analyzed using DeCyder 2-D Differential Analysis software.

### Conclusions

The new Ettan DIGE protocol for cell surface protein labeling is rapid, simple to use and highly specific for labeling cell surface proteins. Many novel cell surface proteins are only detectable when using the cell-surface protein labeling protocol. Over 80 new cell-surface proteins for CHO cells were detected using DeCyder 2-D Differential Analysis Software. Over 80% of the identified cell surface labeled spots were membrane associated proteins.

Multiplexing is achieved using the three CyDye DIGE Fluor minimal dyes, and in combination with DeCyder 2-D software, this new protocol is a powerful tool for studying cell surface proteins with all the advantages obtained with the 2-D DIGE technology.
